# The improvement effect and mechanism of traditional Chinese medicine on chemotherapy-induced diarrhea: a systematic review

**DOI:** 10.3389/fphar.2025.1648107

**Published:** 2026-01-07

**Authors:** Yuzheng Wu, Xiaoyan Cao, Lequan Yu, Jingchun Yao, Chenghong Sun, Jingya Ruan, Dan Wang, Yi Zhang, Tao Wang

**Affiliations:** 1 State Key Laboratory of Component-based Chinese Medicine, Tianjin University of Traditional Chinese Medicine, Tianjin, China; 2 Tianjin Key Laboratory of TCM Chemistry and Analysis, Tianjin University of Traditional Chinese Medicine, Tianjin, China; 3 State Key Laboratory of Integration and Innovation of Classic Formula and Modern Chinese Medicine, Lunan Pharmaceutical Group Co. Ltd., Linyi, China

**Keywords:** chemotherapy-induced diarrhea, traditional Chinese medicine, antioxidant, anti-inflammation, intestinal barrier, gut microbiota

## Abstract

Chemotherapy-induced diarrhea (CID) is one of the gastrointestinal adverse effects in cancer patients after chemotherapy, which seriously affects the course of chemotherapy and reduces the survival rate of patients. The medications commonly used to improve CID include loperamide and octreotide. However, the use of these drugs is often accompanied by adverse effects. Currently, extensive preclinical and clinical studies have demonstrated that specific Traditional Chinese Medicine (TCM) formulations and their active metabolites can effectively alleviate CID. Meanwhile, it has been verified that TCM demonstrates favorable curative outcomes and satisfactory safety, which provides a new alternative for patients suffering from this condition. In this review, we focus on the recent studies in which TCM were applied to treat CID both in animal models and patients, and summarize the mechanism of TCM in improving CID from the aspects of antioxidant, anti-inflammation, maintenance of intestinal mucosal barrier and regulation of intestinal flora. We found that TCM has a definite therapeutic effect in the treatment of CID, which reflected in multiple levels and multiple pathways, including Keap1/Nrf2, TLR4/NLRP3, TLR4/NF-κB, JAK2/STAT3, Wnt, AKT, and MAPK. The aim of this review was to provide some references for applying TCM to treat CID in clinic, and provide new insights into the critical path ahead of the development of innovative drugs in alleviating CID.

## 1 Introduction

The incidence of cancer has shown a significant increase in recent years. It is reported that the number of cancer patients worldwide will reach 28.4 million in 2040 (Sung et al., 2021). Chemotherapy is widely utilized as a prominent anti-cancer method. However, the adverse effects of chemotherapeutic agents on various organ systems, particularly on the gastrointestinal system, draw significant concern. This is primarily because rapidly dividing cells within the gastrointestinal epithelium serve as primary targets for these agents (Novak et al., 2022; Singh et al., 2023). Chemotherapy-induced diarrhea (CID) represents one of the most frequent gastrointestinal adverse effects induced by chemotherapeutic agents. Clinical studies have found that 40%–80% of cancer patients suffer from CID, which results in weight loss, watery stools, intestinal epithelial inflammation, and intestinal villus atrophy (Akbarali et al., 2022; Huang et al., 2022). CID not only damages the intestinal tract and reduces patient quality-of-life, but also result in cessation of treatment and could be life threatening.

The pathogenesis of CID is multifactorial, originating primarily from the cytotoxic impact of chemotherapeutic agents on the rapidly proliferating epithelial cells of the intestinal mucosa (Andreyev et al., 2014). The initial insult often involves oxidative stress, whereby chemotherapeutic agents disrupt enterocyte metabolism, leading to excessive generation of reactive oxygen species (ROS). This oxidative damage subsequently exacerbates injury to intestinal epithelial cells and promotes cell death. A key consequence of this damage is the compromise of epithelial barrier integrity. Increased intestinal permeability facilitates the translocation of luminal toxins and microbes, which in turn activates the mucosal immune system. This triggers a robust inflammatory response, characterized by immune cell infiltration and the release of pro-inflammatory cytokines. Concurrently, the direct damage to intestinal crypt cells impairs the normal cycle of epithelial cell renewal and differentiation, leading to villous atrophy and crypt hyperplasia. This results in a disruption of the critical balance between intestinal absorption and secretion. Additionally, the cumulative structural and functional damage to the gut epithelium, along with the inflamed mucosal environment, creates a dysbiotic state that disrupts the homeostasis of the gut microbiota. This dysbiosis can further impair barrier function, modulate immune responses, and influence intestinal secretion (Benson et al., 2004). Therefore, given this interconnected pathology, therapeutic strategies that can simultaneously target these pathways are needed to effectively manage CID.

**FIGURE 1:**
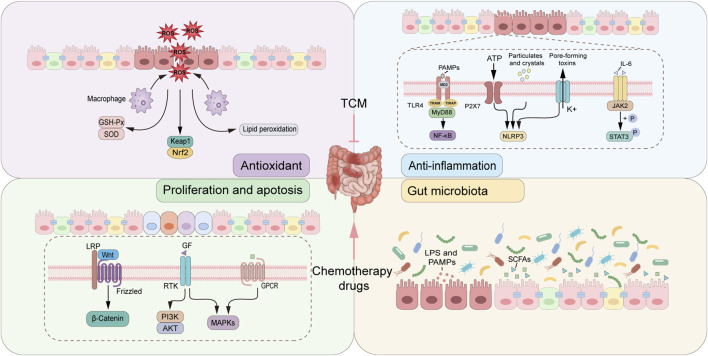
The mechanism of TCM in alleviating CID. TCM improving CID via multiple mechanisms, including reducing oxidative stress products and activating antioxidant signaling pathways, inhibiting inflammatory signaling pathway and reducing the production of proinflammatory factors, inhibiting the apoptosis of epithelial cells, and restoring the homeostasis of intestinal microecology.

At present, loperamide, montmorillonite powder, octreotide, and probiotics are commonly used to improve CID (Thomsen and Vitetta, 2018; Chang et al., 2020). However, despite the therapeutic benefits of these medications, diarrhea and intestinal injury following chemotherapy in cancer patients remains an unsolved clinical issue. Therefore, it is urgent to find alternative drugs with little adverse reactions and good therapeutic efficacy. In recent years, studies have shown that traditional Chinese medicine (TCM) plays an important role in treating adverse reactions caused by chemotherapy, especially gastrointestinal adverse reactions, which can significantly improve nausea, vomiting, diarrhea, and constipation of patients (Zhang et al., 2018; Liu YQ. et al., 2021). Nowadays, clinicians and patients are pursuing the possibility of TCM in alleviating CID to relieve the discomforts of gastrointestinal tract. The TCM used to treat CID usually has the functions of tonifying deficiency, regulating dampness, clearing heat, and strengthening gastrointestinal tract (Chen et al., 2021; Zhang et al., 2021). And accumulating evidences of pharmacological research have suggested that TCM and its active ingredients may play their therapeutic roles by inhibiting intestinal oxidative stress, suppressing inflammation, regulating the proliferation of intestinal epithelial cells, repairing intestinal barrier function and regulating gut microbiota (Fu et al., 2018; Sauruk da Silva et al., 2021; Li et al., 2022). This review systematically synthesized literature retrieved from PubMed and Web of Science (2019–2025) using a search strategy based on key words such as “chemotherapy-induced diarrhea,” “botanical medicine,” and “traditional Chinese medicine.” The inclusion was limited to original research elucidating the mechanisms of TCM interventions for CID, excluding reviews and non-relevant publications. It is hoped that this work will offer novel perspectives to guide research priorities in developing new therapies for CID (Figure 1).

## 2 TCM targeting oxidative stress for management of CID

### 2.1 A brief overview of oxidative stress mechanisms associated with CID

**FIGURE 2:**
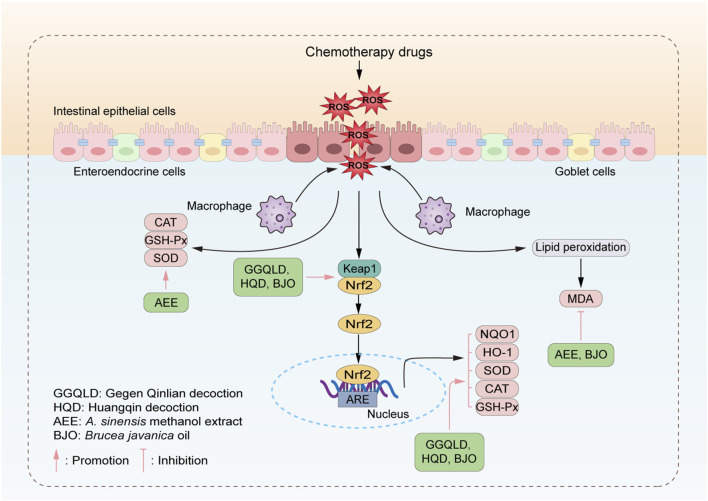
TCM alleviates CID through antioxidant effects. TCM reduces oxidative damage to the intestine via inhibiting oxidation-related pathways, such as downregulating the production of ROS and activating the Keap/Nrf2 signaling pathway. The representative TCMs acting on each pathway are listed in the green boxes.

Oxidative stress refers to the imbalance between oxidants and antioxidants production, which results in the production of a large amount of ROS and reactive nitrogen species (RNS), and inhibition of the activity of antioxidant enzymes, such as glutathione peroxidase (GSH-PX), catalase from *micrococcus* lysodeiktic (CAT), and superoxide dismutase (SOD) (O'Reilly et al., 2024). The accumulation of ROS and RNS results in the oxidation of biological molecules such as DNA, protein, and lipids, which leads to oxidative damage in cells, tissues, or organs (An et al., 2024). Major intracellular signaling pathways regulating redox balance include Keap1/Nrf2, PI3K/AKT, and MAPK. Among these, the Keap1/Nrf2 pathway represents a central mechanism for cellular defense against oxidative stress. Under homeostatic conditions, Keap1 binds to Nrf2, promoting its ubiquitination and proteasomal degradation. Upon exposure to chemotherapy induced oxidative stress, modification of cysteine residues in Keap1 leads to Nrf2 release, stabilization, and nuclear translocation. Within the nucleus, Nrf2 binds to antioxidant response elements (ARE), initiating transcription of cytoprotective genes such as SOD, GSH-Px, NADPH quinone dehydrogenase 1 (NQO1), and heme oxygenase 1 (HO-1) (Zhang et al., 2020). In the specific context of CID, Nrf2 plays a critical yet complex role. Chemotherapeutic agents, including irinotecan and 5 fluorouracil, directly provoke oxidative injury within the intestinal mucosa, depleting antioxidants and disrupting fluid and electrolyte homeostasis, thereby initiating diarrhea (Rtibi et al., 2017). In this setting, Nrf2 activation is primarily protective. Moderate upregulation of Nrf2 signaling attenuates chemotherapy induced damage by enhancing antioxidant capacity, restoring intestinal barrier function through tight junction protein expression, and inhibiting pro inflammatory cytokine production. These actions collectively alleviate diarrheal severity. However, Nrf2 activation can exert dual effects. Sustained or excessive activation may dysregulate immune responses and promote fibrotic processes, potentially impairing mucosal repair. This phenomenon is also observed in inflammatory bowel disease (IBD), highlighting the pathway’s contextual complexity (Geertsema et al., 2023). Consequently, the Keap1/Nrf2 pathway represents a promising yet complex therapeutic target for CID, requiring finely tuned modulation to achieve clinical benefit. Notably, TCM, with its multi-metabolite and system-level approach, has shown potential in preclinical studies to ameliorate chemotherapy-induced intestinal injury through regulation of this pathway. Several botanical drug formulations and bioactive metabolites have been found to activate Nrf2-mediated antioxidant responses, reduce oxidative stress, suggesting a complementary approach for managing CID. Further investigation into the role of TCM in finely regulating the Keap1/Nrf2 axis may provide novel therapeutic avenues for alleviating CID while minimizing side effects (Figure 2).

### 2.2 TCM prescriptions with antioxidant effects

Gegen Qinlian decoction (GGQLD), as documented in Shang Han Lun by Zhang Zhongjing during the Han dynasty, is a renowned TCM formula utilized for treating diarrhea. It is comprised *Pueraria lobata* (Willd.) Ohwi, *Scutellaria baicalensis* Georgi, *Coptis chinensis* Franch., and *Glycyrrhiza uralensis* Fisch., and widely employed in contemporary clinical practice to effectively manage various gastrointestinal disorders, particularly diarrhea. A clinical study has shown that, treatment of GGQLD for 4 courses (14 days each course) could significantly reduce diarrhea rate for 2 fold (47.37% vs. 23.08%, *P* < 0.05) of the patients who receive FOLFOX chemotherapy regimens (oxaliplatin and calcium folinic acid and 5-FU) (Haiming et al., 2023). While this result is promising, these findings are limited by the study design. The treatment group received GGQLD as an add-on to montmorillonite powder. This design introduces a confounding factor, making it impossible to determine whether the benefit came from GGQLD alone or from its combination with montmorillonite powder. Beyond the clinical findings, the therapeutic potential of GGQLD is further supported by mechanistic investigations in animal models. Wu et al. have found that GGQLD could ameliorate CPT-11 induced-diarrhea in mice, reverse the loss of body weight, and restore the damage of colon. Meanwhile, GGQLD could activate Keap1/Nrf2 signaling pathway, promote the expression of HO-1 protein in colon, and increase the GSH-PX activity, suggesting that GGQLD improves CID through antioxidant action (Wu et al., 2021). In general, both clinical and animal studies have demonstrated that GGQLD could alleviate chemotherapy-induced diarrhea, and animal study shown that the therapeutic effect of GGQLD may be related to its antioxidant effect.

### 2.3 TCM and its metabolites with antioxidant effects

#### 2.3.1 Aquilaria sinensis (Lour.) Gilg

*Aquilaria sinensis* (Lour.) Gilg, a resin-rich wood, possesses a variety of TCM effects, including moving qi and relieving pain, warming middle energizer to arrest vomiting, nourishing qi and relieving asthma, has been used to treat diarrhea and fever and play a key role in regulating gastrointestinal function (Hashim et al., 2016). *A. sinensis* methanol extract (AEE) at 3 g/kg was clarified to increase the activity of SOD in mice serum, decrease the level of MDA and reduce the production of ROS in intestinal tract via inhibiting oxidative stress response, thereby attenuating 5-FU induced intestinal mucosal damage and alleviating diarrhea in mice in a dose-dependent manner (Zheng et al., 2019). These results indicated that the underlying mechanism by which AEE improves CID might be associated with its antioxidant effect.

#### 2.3.2 Brucea javanica oil

*Brucea javanica* oil (BJO) is an oil metabolite extracted from the dried ripe fruit of the anti-dysenteric drug *B. javanica* (L.) Merr., which has antioxidant and anti-inflammatory activities. BJO is widely used as an effective adjuvant therapy to relieve the side effects of radiotherapy and chemotherapy (Huang et al., 2017). A clinical study has shown that BJO injection significantly improved diarrhea on Ⅱb-Ⅲb cervical cancer patients during radiotherapy and chemotherapy. After 4 courses of treatment, the incidence of diarrhea was reported to be 33.33% in the BJO injection treated group. However, in the non-treated group, over 60% of Ⅱb-Ⅲb cervical cancer patients suffered from diarrhea (Feihui and Li, 2022). However, the article lacks the necessary details on quality control and chemical standardization for the BJO injection, thereby undermining the reproducibility of the research. Addition to these clinical findings, animal studies provide mechanistic insights into BJO’s action. Animal study has shown that BJO effectively increased the activity of SOD and reduced the concentration of MDA in the serum. Furthermore, BJO inhibited oxidative stress in the ileum by activating the Nrf2/HO-1 signaling pathway, which protected intestinal epithelium cells from oxidative damage and improving 5-FU induced diarrhea (Zheng et al., 2023).

## 3 TCM targeting inflammatory pathways for management of CID

### 3.1 A brief overview of inflammatory pathways associated with CID

**FIGURE 3:**
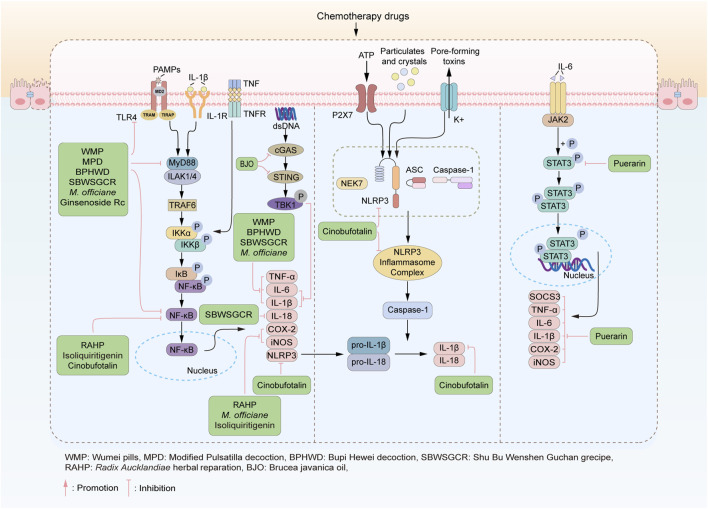
TCM alleviates CID through anti-inflammatory effects. TCM play anti-inflammatory effects via suppressing the production of proinflammatory cytokines, inhibiting TLR-mediated inflammatory response, preventing assembly of NLRP3 inflammasome complex, and inhibiting activation of STAT3. The representative TCMs acting on each pathway are listed in the green boxes.

Chronic inflammation and associated signaling pathways are critically involved in cancer development and progression. Sustained activation of pathways such as Janus kinase 2/signal transducer and activator of transcription 3 (JAK2/STAT3) and Toll-like receptor 4 (TLR4) promotes tumor proliferation, invasion, and metastasis. For instance, IL-6/JAK/STAT3 signaling enhances bladder cancer cell growth and is linked to targeted kinase inhibitor resistance (Gao et al., 2023), while TLR4 activation facilitates inflammation-associated carcinogenesis and liver cancer metastasis through protein tyrosine kinase 2 (PTK2) induction (Feng et al., 2023). Within the context of CID, intestinal inflammation serves as a key driver of pathogenesis. Preclinical evidence indicates that chemotherapy drugs damage intestinal mucosal cells, activating the immune system and triggering the release of inflammatory mediators (Lee et al., 2014). Disruption of the mucus layer permits bacterial contact with epithelia, activating toll-like receptors and upregulating nuclear factor-κB (NF-κB) and NOD-like receptor thermal protein domain associated protein (NLRP) signaling, which amplifies pro-inflammatory cytokine production (Reuven et al., 2014; Abraham et al., 2022). Preclinical studies indicate that intestinal inflammation impairs absorptive function, reduces electrolyte uptake, increases luminal osmotic pressure, and stimulates excessive mucus and fluid secretion, which finally result in diarrhea (Akbarali et al., 2022). Inflammatory activation of the enteric nervous system further accelerates intestinal transit and fluid secretion, exacerbating diarrheal symptoms (Spiller, 2002). Given the central role of inflammation in CID, targeting these pathways offers a promising therapeutic strategy. TCM has demonstrated potential in preclinical models to alleviate intestinal inflammation by modulating NF-κB, TLR4, JAK/STAT, and NLRP3 pathways, reducing pro-inflammatory cytokines, and restoring mucosal integrity. It is important to note, however, that these mechanistic insights are derived primarily from animal studies and *in vitro* experiments. While these findings position TCM as a promising complementary approach for CID management through anti-inflammatory mechanisms (Figure 3), further clinical trials are warranted to substantiate its efficacy and safety in human patients.

### 3.2 TCM prescriptions with anti-inflammatory effects

#### 3.2.1 Wumei pills

The prescription of Wumei pills (WMP) derived from Shang Han Lun is composed of *Prunus mume* (Sieb.) Sieb. et Zuce, *Asarum heterotropoides* Fr. Schmidt var. *mandshuricum* (Maxim) Kitag, *Zingiber officinaie* Rosc., *C. chinensis*, *A. sinensis*, *Aconitum carmichaelii* Debx., *Zanthoxylum bungeanum* Maxim., *Cinnamomum cassia* Presl, *Panax ginseng* C. A. Mey., and *Phellodendron chinense* Schneid. A clinical study has recruited 50 CID patients differently treated with loperamide or WMP. After 4 weeks of treatment, the incidence of diarrhea was observed. The results showed that the diarrhea rate of WMP group was 8%, significantly lower than that of loperamide group (52%). It was also found that the serum levels of proinflammatory cytokines IL-1β, IL-6, and TNF-α in WMP group were decreased, and the level of anti-inflammatory cytokine IL-10 was increased (Dongxue et al., 2021). However, this finding must be interpreted with caution due to the relatively small sample size (n = 50), which may limit the statistical power and generalizability of the results. The therapeutic potential of WMP is further supported by mechanistic insights from preclinical studies. For instance, study has found that WMP at the dosage of 11.325 g/kg inhibited the expressions of TNF-α, IL-1β and IL-6 in mouse jejunum and colon by blocking TLR4/MyD88/NF-κB signaling pathway. Meanwhile, it was found to inhibit the inflammatory response by reducing the infiltration of neutrophil into the intestinal tissue. In addition, WMP could promote the expressions of Zonula occluden 1 (ZO-1), Claudin-1 and E-cadherin, increase the synthesis and secretion of Mucin-2, restores the integrity of intestinal mucosal barrier, which in turn reducing the intestinal mucosal injury and improving 5-FU-induced diarrhea (Lu et al., 2022).

#### 3.2.2 Modified Pulsatilla decoction

Modified Pulsatilla decoction (MPD), a classical formula documented in Zhang Zhongjing’s Synopsis of the Golden Chamber from the Han Dynasty, is specifically indicated for severe deficiency of qi and blood complicated by dysentery. This formulation integrates *Pulsatilla chinensis* (Bge.) Regel, *C. chinensis Franch.*, *P. chinense* Schneid., *Fraxinus rhynchophylla* Hance with *G. uralensis* Fisch. and *Asini Corii Colla*, exhibiting multi-dimensional therapeutic properties including heat-clearing, dysentery-arresting, qi-tonifying, and blood-nourishing capacities. Study has demonstrated that MPD significantly ameliorated 5-fluorouracil (5-FU)-induced symptoms in murine models, including diarrhea and hematochezia. Molecular docking analysis revealed high-affinity binding of its bioactive constituents, pulsatilla saponin B4, glycyrrhizic acid, and limonin, to critical inflammatory proteins (TLR4, MyD88, TRAF6, NF-κB), effectively suppressing TLR4/MyD88/NF-κB pathway initiation. Western blot analysis further confirmed marked suppression of TLR4, MyD88, and NF-κB protein expression by the MPD. Consequently, this inhibition substantially reduced levels of pro-inflammatory mediators (LPS, IL-1β, TNF-α, IFN-γ, IL-6, IL-8, IL-17) while elevating anti-inflammatory IL-10 in 5-FU-challenged mice (Qiu et al., 2025).

#### 3.2.3 Bupi Hewei decoction

Clinical studies have confirmed that Bupi Hewei decoction (BPHWD), which is composed of *Astragalus membranaceus* (Fisch.) Bge. var. *mongholicus* (Bge.) Hsiao, *A. macrocephala*, *Citrus aurantium* L., *Pinellia ternata* (Thunb.) Breit., Massa Medicata Fermentata (MMF), *Hordeum vulgare* L., and *P. cocos*, has the potential to improve diarrhea and vomiting caused by chemotherapy (Sun et al., 2020). Sun et al. found that BPHWD might inhibit the over expressions of TNF-α, IL-1β and IL-6 by blocking the activation of TLR4/NF-κB signaling pathway and enhance the expressions of ZO-1 and E-cadherin, thereby reducing intestinal mucosal inflammation caused by 5-FU and alleviating diarrhea in rats (Sun et al., 2019).

#### 3.2.4 Shubu Wenshen Guchang recipe

Shubu Wenshen Guchang recipe is a TCM prescription prepared by Professor Wang Renqiang, which is composed of *Bupleurum chinense* DC., *Perilla frutescens* (L.) Britt., *C*. *aurantium*, *Pseudostellaria heterophylla* (Miq.) Hoffm., *A. macrocephala*, *Morinda officinalis* How., *Epimedium brevicornu* Maxim., *Curculigo orchioides* Gaertn. *Terminalia chebula* Retz., *Rosa laevigata* Michx., and *Aucklandia lappa* Decne. It has the effect of strengthening spleen, kidney, and intestine (Qiong and Dingyu, 2014). Zhang et al. have established an animal model of delayed diarrhea induced by CPT-11. They have found that the symptoms of CID were significantly improved after 5 days of treatment with this formula. In addition, the expressions of pro-inflammatory cytokines IL-1β, IL-18 and TNF-α, as well as mRNA and protein expression levels of TLR4 and NF-κB p65 in mouse intestinal tissues were downregulated, suggesting that this formula might inhibit the activation of TLR4/NF-κB signaling pathway, whereby alleviating CPT-11 induced delayed diarrhea (Zhang Q. et al., 2022).

#### 3.2.5 Radix Aucklandiae botanical drug preparation

According to the record of TCM, the Radix Aucklandiae botanical drug preparation (RABD) has the effect of tonifying qi and strengthening spleen and stomach. The composition of RABD is based on the classic formula Liujunzi decoction with the addition of *A. lappa* and *Amomum villosum* Lour. Study has shown that RABD played a role in protecting the gastrointestinal tract and improving CID (Xiao et al., 2012). And Liu et al. has revealed that the RABD improved diarrhea induced by 5-FU via reducing the expressions of COX-2 and NF-κB p65 in jejunum and colon tissue, and inhibiting the expressions of IL-6, IL-1β and TNF-α in mouse serum. Additionally, RABD also restored the villus height and crypt depth of mice, promoted the proliferation of goblet cells, and repaired intestinal mucosal barrier. These results indicated that the therapeutic effect of RABD on CID might be associated with its anti-inflammatory effect (Liu JH. et al., 2021).

### 3.3 TCM and its metabolites with anti-inflammatory effects

#### 3.3.1 Brucea javanica oil

In clinical practice, Brucea javanica oil (BJO) is predominantly co-administered with CPT-11 or docetaxel to potentiate their anticancer efficacy. It is noteworthy that BJO significantly enhanced the response rate in lung cancer patients with brain metastases, while concomitantly preserving immune function and improving quality of life. Critically, co-administration of BJO and anticancer drugs demonstrated a marked reduction in chemotherapy-induced gastrointestinal toxicity (Shi et al., 2022). Experimental investigations have established that BJO effectively antagonizes CPT-11-induced activation of the cyclic GMP-AMP synthase-stimulator of interferon genes (cGAS-STING) pathway in murine intestinal tissue at the dosage of 0.25, 0.5, and 1.0 g/kg. This suppression subsequently attenuated secretion of IL-1β and IL-6 and restored intestinal barrier integrity. Crucially, when co-administered with the STING agonist DMXAA, BJO-mediated inhibition of cGAS, STING, phosphorylated TANK-binding kinase 1 (p-TBK1), and phosphorylated interferon regulatory factor 3 (p-IRF3) was abrogated. This pharmacological reversal mechanistically validates that BJO ameliorates CID through targeted suppression of cGAS-STING signaling axis activation (Lai et al., 2025).

#### 3.3.2 Ginsenoside Rc

**FIGURE 4:**
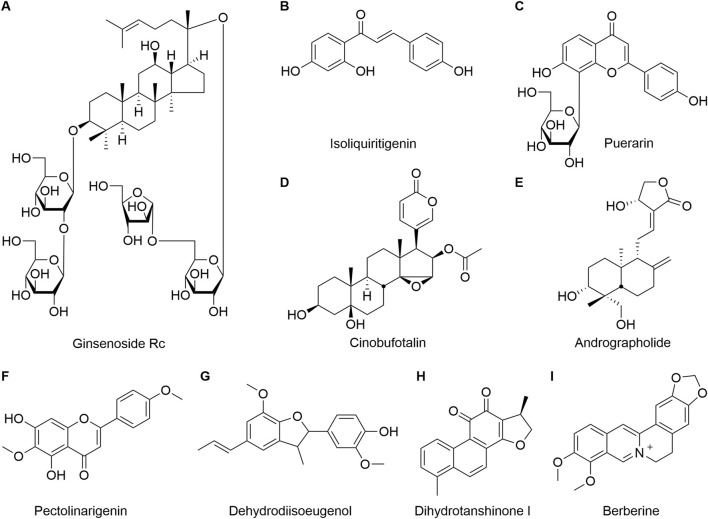
The structures of the metabolites mentioned in the article. **(A)** Ginsenoside Rc; **(B)** Isoliquiritigenin; **(C)** Puerarin; **(D)** Cinobufotalin; **(E)** Andrographolide; **(F)** Pectolinarigenin; **(G)** Dehydrodiisoeugenol; **(H)** Dihydrotanshinone I; **(I)** Berberine.

Ginsenoside Rc (G-Rc, Figure 4A), a predominant bioactive constituent of *P. ginseng* C. A. Mey., exhibits potent anti-inflammatory and antioxidant activities. Previous evidence demonstrated that G-Rc ameliorates dextran sulfate sodium (DSS)-induced ulcerative colitis by suppressing intestinal inflammation and restoring barrier dysfunction. Notably, Xu et al. validated its efficacy in mitigating 5-FU-associated diarrhea, concurrently alleviating anticancer drugs induced enteritis and barrier injury. Network pharmacology and molecular docking analyses revealed high-affinity binding of G-Rc to pivotal nodes within CID-related pathways, including PI3K-AKT, chemokine signaling, and NF-κB cascades. Further, subsequent *in vivo* and *in vitro* validation confirmed G-Rc mediated suppression of the PI3K-AKT/NF-κB signaling axis (Xu et al., 2024).

#### 3.3.3 Isoliquiritigenin

*G. uralensis* is a commonly used medicine for the treatment of gastrointestinal diseases in TCM. The main flavonoid, isoliquiritigenin (Figure 4B) in it was reported to protect gastrointestinal mucosa from being damaged by inflammatory response (Zhang Z. et al., 2022). Liao et al. have found that isoliquiritigenin inhibited the activation of NF-κB and reduced the expressions of prostaglandin-endoperoxide synthase 2 (PTGS2), TNF-α, and nitric oxide synthase 2 (NOS2) in the colon tissues of 5-FU-induced CID mice, as well as repaired the integrity of intestinal mucosal barrier through restoring the height of intestinal villi and alleviating edema in intestinal epithelium (Liao et al., 2022).

#### 3.3.4 Puerarin

*P. lobata* is widely used in TCM to treat gastrointestinal diseases. And puerarin (Figure 4C), one of the main active metabolites of *P. lobata*, has excellent anti-inflammatory effect (Ma et al., 2021). A pharmacological study has found that puerarin had the potential to ameliorate 5-FU-induced diarrhea by inhibiting the phosphorylation of Janus kinase 2 (Jak2), blocking the activation of signal transducer and activator of transcription 3 (Stat3) and suppressing the expression of its downstream proinflammatory cytokine suppressor of cytokine signaling 3 (SOCS3). Furthermore, the study also shown that puerarin inhibited the expressions of COX-2, iNOS, IL-1β, IL-6 and TNF-α in colon tissue of mice, thereby alleviating intestinal mucosal injury induced by 5-FU and improving diarrhea in mice (Wang et al., 2021).

#### 3.3.5 Cinobufotalin

Cinobufotalin (Figure 4D) is an active ingredient obtained from the skin of Chinese toad *Bufo gargarizans* Cantor, which has anti-tumor, immune regulatory and anti-inflammatory effect. Study has shown that cinobufotalin in combination with chemotherapy agents could be used to improve the efficacy of anti-tumor drugs as well as alleviate the diarrhea caused by them (Yingquan et al., 2022). Li et al. had reported that cinobufotalin reduced the excessive secretion of serum inflammatory factors IL-6, IL-1β and TNF-α by inhibiting the activation of NF-κB/NLRP3 signaling pathway. Furthermore, the study also revealed that it suppressed the levels of diamine oxidase (DAO), endotoxin (ET) and D-lactic acid (D-LA) in mice, and promoted the expressions of occludin and claudin-1 in small intestinal, which enhanced the intestinal mucosal barrier function, and thus improve the intestinal damage and diarrhea caused by the treatment of FOLFOX chemotherapy regimens (Shichao et al., 2022).

## 4 TCM targeting cell proliferation and apoptosis pathways for management of CID

### 4.1 A brief overview of cell proliferation and apoptosis pathways associated with CID

**FIGURE 5:**
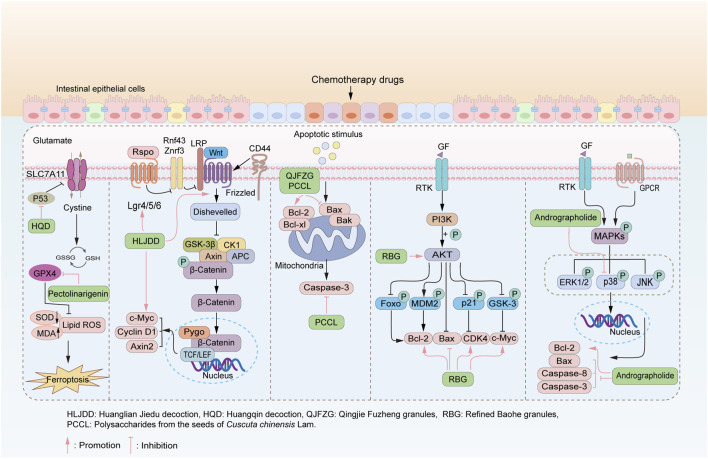
TCM alleviates CID through regulating cell proliferation and apoptosis. TCM protects the intestine by regulating the expressions of Bcl-2, Bax, Caspase-3, and Caspase-8, activating Wnt and AKT signaling pathways, and inhibiting the phosphorylation of p38 MAPK. The representative TCMs acting on each pathway are listed in the green boxes.

CID arises in part from excessive apoptosis of intestinal epithelial cells, which is an unintended consequence of chemotherapy that damages the intestinal mucosa alongside tumor cells. This abnormal enterocyte apoptosis leads to villous atrophy, crypt destruction, and impairment of the mucosal barrier, resulting in malabsorption of water and electrolytes and ultimately diarrhea (Hamouda et al., 2017). The execution of apoptosis is primarily regulated through key signaling pathways including protein kinase B (PKB, also known as AKT), mitogen-activated protein kinase (MAPK) and Wnt signaling pathways, which are activated by chemotherapy-induced DNA damage. These pathways converge on the activation of caspase cascades, leading to DNA fragmentation, cytoskeletal degradation, and nuclear dysfunction (Kwak et al., 2023; Yang et al., 2023). Notably, caspase-3 overexpression disrupts epithelial integrity and hinders repair (Kuo et al., 2019), while caspase-1 activation promotes secretion of IL-1β and IL-18, amplifying inflammation and tissue injury (Lv et al., 2021). Another important protein family involved in apoptosis is B-cell lymphoma-2 (Bcl-2). The Bcl-2 protein can form heterodimers with pro-apoptotic proteins such as Bax, thereby inhibiting the functions of pro-apoptotic proteins and preventing cell apoptosis (Cory and Adams, 2002). Beyond apoptosis, ferroptosis—an iron-dependent cell death process driven by GPX4 suppression, lipid peroxidation, and glutathione depletion—also contributes significantly to epithelial damage and barrier failure in CID (Xu et al., 2021). Study has shown that inhibition of ferroptosis through regulating iron load and GPX4 expression significantly alleviates epithelial cell death and promotes recovery in colitis models (Wang et al., 2023). Clinical evidence further supports its pathophysiological relevance, with studies demonstrating reduced GPX4 expression in colon tissues from patients with intestinal barrier injury, where lower levels correlate with more severe disease (Sun et al., 2025). Activation of these cell death pathways increases intestinal permeability, facilitating toxin penetration and bacterial translocation, which exacerbates diarrhea (Hueso et al., 2020). Targeting apoptotic and ferroptosis pathway has thus emerged as a rational strategy for CID management. In this context, TCM has shown promise in preclinical studies for its ability to inhibit epithelial apoptosis, modulate ferroptosis, promote proliferation, and enhance barrier function, thereby potentially alleviating CID (Figure 5). It should be noted, however, that these mechanisms are derived primarily from animal and cellular studies, and clinical efficacy remains to be firmly established through well-controlled trials in human patients.

### 4.2 TCM prescriptions modulate cell proliferation and apoptosis

#### 4.2.1 Huanglian Jiedu decoction

Huanglian Jiedu decoction (HLJDD) is composed of *C. chinensis*, *P. chinense*, *Gardenia jasminoides* Ellis and *S. baicalensis*, which has the effect of clearing heat and detoxifying (Chen et al., 2022). To investigate the therapeutic effect of HLJDD on CID, Chan et al. established a rodent model of diarrhea induced by 5-FU and CPT-11 and evaluated the preparation at doses of 50, 100, and 200 mg/kg. And found that HLJDD significantly improved the symptoms of diarrhea in mice, decreased the number of TUNEL positive cells, increased the number of Ki67 positive cells, and suppressed the expression of Caspase-3 in jejunum tissue of mice. Moreover, HLJDD was found to increase crypt cell proliferation-related protein CD44 and stem cell regeneration related protein Leucine repeat G protein-coupled receptor 5 (Lgr5) in intestinal tissue, further suggesting that HLJDD activated the Wnt signaling pathway, whereby promoting the renewal and regeneration of intestinal epithelial cells, and then alleviated the diarrhea of mice (Chan et al., 2020).

#### 4.2.2 Huangqin decoction

Huangqin Decoction (HQD), originating from Shang Han Lun, is composed of four botanical drug metabolites: *S. baicalensis* Georgi, *G. uralensis* Fisch., *Paeonia lactiflora* Pall., and *Ziziphus jujuba* Mill. HQD has been reported to effectively alleviate CPT-11-induced intestinal toxicity while concurrently enhancing the anticancer efficacy of CPT-11 (Cui et al., 2017). Clinical studies demonstrated that HQD can effectively mitigate chemotherapy-induced intestinal injury, preventing and treating CID without compromising the anticancer effects of chemotherapeutic agents (Yang et al., 2015). However, there were some limitations in the study design. While the study employed randomization, it fails to mention the implementation of blinding. The absence of blinding for participants, clinicians, and outcome assessors severely compromises the result integrity, as it creates a high risk of performance and detection bias, especially given the reliance on subjective patient-reported outcomes. While these methodological concerns exist, compelling mechanistic evidence from preclinical studies supports HQD’s therapeutic potential. Wang et al. established a CID mouse model using CPT-11 induction and subsequently administered HQD as treatment. Experimental results revealed that HQD significantly reduced the diarrhea score in CID model mice, ameliorated inflammatory pathological damage, and decreased serum levels of IL-1β and TNF-α. Furthermore, HQD restored intestinal barrier function and upregulated the expression of the tight junction proteins tight junction protein 1 (TJP-1) and occludin. Additionally, HQD significantly inhibited intestinal epithelial cell ferroptosis by ameliorating iron deposition and suppressing reactive oxygen species (ROS) generation. Network pharmacology screening implicated p53 as a key target mediating HQD-mediated inhibition of ferroptosis and amelioration of CID. Subsequent analysis of p53, SLC7A11, and GPX4 expression in murine intestinal tissue demonstrated that CPT-11 administration elevated p53 levels while reducing SLC7A11 and GPX4 levels. However, HQD treatment significantly reversed the CPT-11-induced increase in intestinal p53 and the decrease in SLC7A11 and GPX4 (Wang et al., 2025). These results indicated that HQD prevent CID by activating the p53/SLC7A11/GPX4 signaling pathway, inhibiting ferroptosis, and alleviating intestinal inflammatory injury.

#### 4.2.3 Qingjie Fuzheng granules

Qingjie Fuzheng granules (QJFZG) is composed of *Scleromitrion diffusum* (Willd.) R. J. Wang, *H. vulgare*, *A. memeranaceus* var. mongholicus and *Scutellaria barbata* D. Don. It has the effect of clearing heat, detoxifying qi and strengthening spleen (Zhong et al., 2018). It is not only often used in combination with chemotherapy drugs to treat cancer, but also has therapeutic effects in the treatment of CID. Zhang et al. have found that QJFZG downregulated the expressions of pro-apoptotic proteins Bax and p21, upregulated the expressions of anti-apoptotic protein Bcl-2, and reduced the number of TUNEL positive cells in mouse jejunum. Meanwhile, QJFZG was clarified to promote cell proliferation in jejunum tissue of mice through increasing the expressions of proliferating cell nuclear antigen (PCNA), Cyclin D1 and CDK4. Therefore, the injury of intestinal barrier induced by 5-FU was repaired, and diarrhea was improved (Zhang et al., 2019).

### 4.3 TCM and its metabolites modulate cell proliferation and apoptosis

#### 4.3.1 Polysaccharides from the seeds of Cuscuta chinensis Lam

Ji et al. have explored the therapeutic effect of polysaccharides from the seeds of *Cuscuta chinensis* Lam. (PCCL) in 5-FU induced diarrhea in mice. The results revealed that PCCL repaired intestinal villi and crypt structure in jejunum. Moreover, PCCL decreased the number of TUNEL positive cells in jejunum, inhibited the expressions of Caspase-3 and Bax, and upregulated the expression of Bcl-2, which indicated that PCCL had the potential to alleviate CID through regulating intestinal cell proliferation and apoptosis (Ji et al., 2022).

#### 4.3.2 Andrographolide

Andrographolide (Figure 4E) is the main active metabolite of *Andrographis paniculata* (Burm. f.) Nees, which has anti-bacterial and anti-inflammatory effects. Study has shown that andrographolide could effectively relieve diarrhea in mice with colitis (Gao et al., 2018). Xiang et al. has reported that andrographolide could significantly decrease the number of TUNEL positive cells in the ileum and colon of 5-FU-induced intestinal mucositis in mice, increase the expression of PCNA, block the phosphorylation of p38 MAPK, downregulate the expression of Bax, and upregulate the expression of Bcl-2. Furthermore, the expressions of Caspase-8 and Caspase-3 in ileum were inhibited by the treatment of andrographolide, and the integrity of intestinal villi and crypt structure was restored (Xiang et al., 2020). These results suggested that andrographolide might regulate the apoptosis and proliferation of intestinal epithelial cells by inhibiting the activation of p38 MAPK signaling pathway, which promoted the repair of intestinal mucosal damage, and thus alleviated diarrhea in mice.

#### 4.3.3 Pectolinarigenin

Pectolinarigenin (Figure 4F), a flavonoid isolated from the traditional Chinese medicine *Cirsium japonicum* Fisch. ex DC., exhibits anti-inflammatory, antioxidant, and anticancer properties. Pectolinarigenin has been reported to inhibit colon cancer cells in both *in vivo* and *in vitro* models (Gan et al., 2019). Previous studies demonstrated that pectolinarigenin exerts anti-inflammatory and antioxidant effects by elevating levels of GSH and superoxide dismutase (SOD), significantly ameliorating various inflammatory diseases and organ injuries (Feng et al., 2022). A recent study evaluated the therapeutic effects of pectolinarigenin on CID in both intestinal epithelial cells and a CID mouse model. The results indicated that pectolinarigenin significantly ameliorates CID by inhibiting iron deposition, reducing ROS generation, increasing GSH synthesis, and suppressing 5-FU-induced ferroptosis in intestinal epithelial cells.

Transcriptomic and Kyoto Encyclopedia of Genes and Genomes (KEGG) pathway analyses suggested that the PPARγ/GPX4 signaling pathway plays a critical role in the protective effect of pectolinarigenin against 5-FU-induced intestinal epithelial cell injury. Western blot analysis of colon tissues from pectolinarigenin-treated mice revealed that pectolinarigenin significantly suppressed ferritin heavy chain (FTH) protein levels while increasing PPARγ and GPX4 protein levels. These findings indicate that pectolinarigenin significantly alleviates CID by activating the PPARγ/GPX4 axis. Moerover, suppressing 5-FU-induced intestinal epithelial cell ferroptosis represents a promising therapeutic strategy for CID mitigation.

## 5 TCM targeting gut microbiota for management of CID

### 5.1 A brief overview of gut microbiota dysregulation associated with CID

**FIGURE 6:**
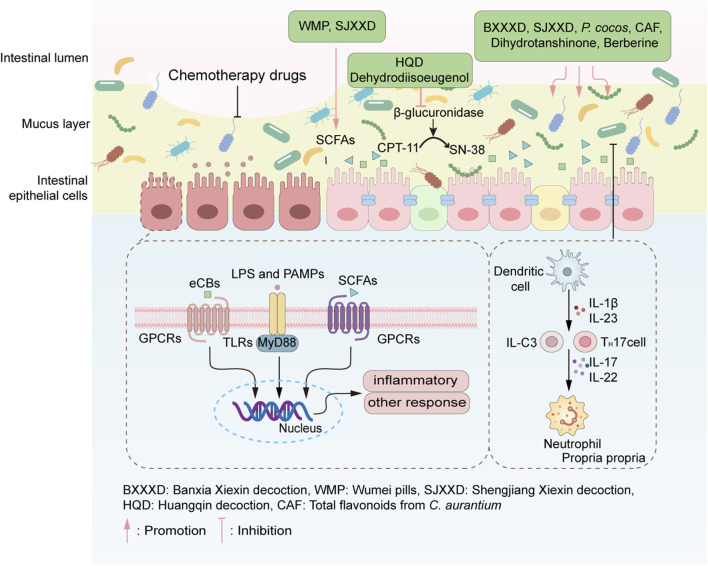
TCM alleviates CID through restoring the homeostasis of intestinal microecology. TCM regulating the intestinal microecology through increasing the abundance and diversity of the gut microbiota, as well as promoting the production of SCFAs. A healthy intestinal microecology strengthened the intestinal mucous layer, prevented intestinal epithelium from being exposed to microorganisms, thus reducing the immune inflammatory response. The representative TCMs acting on each pathway are listed in the green boxes.

Dysregulation of the gut microbiota is a well-established feature of CID (Bauer and Greten, 2023). Clinical studies have demonstrated that the gut microbial community in CID patients exhibits structural dysbiosis, characterized by reduced taxonomic diversity and ecological stability compared to healthy individuals (Alexander et al., 2017). For example, researchers conducted 16S rRNA gene sequencing analysis on fecal samples of 28 patients with non-Hodgkin’s lymphoma before and after chemotherapy. The results showed that chemotherapy drugs significantly increased the abundance of *Proteobacteria*, which is one of the main pathogenic bacteria causing diarrhea. Meanwhile, the abundances of *Firmicutes* and *Actinobacteria* were decreased (Montassier et al., 2015). This microbial imbalance profoundly affects the production of bacterial metabolites, particularly short-chain fatty acids (SCFAs) such as acetate, propionate, and butyrate. Under normal physiological conditions, SCFAs serve as the primary energy source for colonocytes, promote epithelial proliferation and differentiation, enhance mucosal barrier function, and support immunoregulation through mechanisms such as Treg cell activation (Arpaia et al., 2013). However, chemotherapy-induced microbiota disruption leads to decreased SCFA production, which compromises intestinal immune homeostasis, impairs epithelial repair, and contributes to diarrhea pathogenesis (Alexander et al., 2017). Additionally, preclinical evidence suggests that SCFAs stimulate sodium and water absorption via mechanisms independent of circulating AMP, and their deficiency may further exacerbate diarrheal symptoms (Lu et al., 2016). Beyond SCFAs, gut microbiota also influences CID through drug metabolism. The gut microbiota can participate in the metabolism of chemotherapy drugs, enabling certain chemotherapy drugs to undergo chemical transformations and increasing their toxicity. For example, the intestinal bacteria β-glucuronidase can convert the inactive metabolite of CPT-11 (SN-38G) into the active toxic metabolite SN-38. These toxic metabolites will damage the intestinal mucosa and increase the risk of diarrhea (Wallace et al., 2010). Given the central role of gut microbiota and their metabolites in CID, strategies aimed at modulating microbial composition and function represent promising therapeutic avenues. Growing preliminary research suggests that TCM may help restore microbial diversity, regulate microbial metabolites, and support barrier function, thereby potentially alleviating CID (Figure 6). However, these mechanisms remain largely derived from preclinical or correlative human studies, and further clinical trials are needed to establish causal efficacy in patients.

### 5.2 TCM prescriptions with gut-microbiota-modulating properties

#### 5.2.1 Banxia Xiexin decoction

Banxia Xiexin decoction (BXXXD) is a classic prescription derived from Shang Han Lun, which is composed of *Pinellia ternate* (Thunb.) Breit., *Zingiber officinale* Roscoe, *C. chinensis*, *S. baicalensis*, *P. ginseng*, *Z. jujuba*, and *G. uralensis*. It is commonly used to treat various gastrointestinal diseases, including chemotherapy induced diarrhea (Wang Y. et al., 2024). He et al. have found that the combinational use of BXXXD and glutamine significantly restored the diversity of intestinal flora in rats with 5-FU-induced diarrhea, significantly increased the abundance of *Firmicutes* and *Patescibacteria*, reduced the abundance of *Bacteroidetes*, which reduce the disturbance of gut microbiota and protected the microbial barrier of gastrointestinal tract (Wenguang et al., 2023).

#### 5.2.2 Wumei pills

WMP not only has the effect of anti-inflammation, but also has the potential to regulate the diversity of gut microbiota. One study has shown that WMP increased the abundance of *Firmicutes* and decreased the abundance of *Bacteroidetes* at the phylum level. Moreover, it increased the abundance of *Lactobacillaceae* and *Bacteroidaceae*, and decreased the abundance of *Staphylococcales* and *Muribaculaceae* at the family level. At the same time, it increased the production of SCFAs, such as acetate, propionate, and butyrate in colon, which played protective roles in repairing intestinal barrier and alleviating 5-FU-induced diarrhea (Lu et al., 2022). In another fecal microbiota transplantation (FMT) study, fresh fecal samples were collected from CID mice treated with WMP and processed into bacterial suspensions. Subsequently, these suspensions were intragastrically administered to another group of CID mice. The results demonstrated that FMT-treated CID mice exhibited significant increases in body weight, food intake, and colon length, accompanied by a reduction in diarrhea score. Furthermore, FMT ameliorated pathological inflammatory cell infiltration, decreased histopathological scores in both the colon and jejunum, reduced the expression levels of IL-1β, myeloperoxidase (MPO), TNF-α, and IL-6, and reversed the activation of the TLR4/MyD88/NF-κB signaling pathway. These findings indicated that WMP improves CID by modulating chemotherapy-induced gut microbiota dysbiosis (Lu et al., 2024).

#### 5.2.3 Huangqin decoction

HQD has been used clinically in China for over 1800 years to treat gastrointestinal disorders. Studie has shown that HQD effectively ameliorates CPT-11-induced delayed-onset diarrhea, suppresses the levels of IL-1β, IL-6, TNF-α, and MPO in mouse colon tissue, repairs the damaged intestinal epithelial barrier, and restores CPT-11-induced gut microbiota dysbiosis. Further research indicates that baicalin, the major active constituent in HQD, not only inhibits the overgrowth of the harmful bacterium *Escherichia coli*, but also suppresses the expression and activity of bacterial β-glucuronidase. This suppression consequently inhibits the metabolic conversion of CPT-11 into SN-38, which is a gastrointestinally toxic metabolite (Teng et al., 2024). These findings not only provide a deeper elucidation of the mechanism by which HQD ameliorates CID, but also offer a theoretical foundation for the molecular structural optimization of β-glucuronidase-targeting agents.

### 5.3 TCM and its metabolites with gut-microbiota-modulating properties

#### 5.3.1 Poria cocos (Schw.) Wolf (Poria)

*P. cocos* has been widely used in many classic TCM prescriptions, such as Shenling Baizhu Powder, Linggui Zhugan decoction and Sijunzi decoction. It has been reported that *P. cocos* could improve intestinal barrier function via regulating gut microbiota (Wu et al., 2019). Zou et al. have explored the therapeutic effect of *P. cocos* on intestinal damage caused by cisplatin. The results showed that *P. cocos* and its water-soluble polysaccharide (WP) could alleviate intestinal damage. Mechanistic studies have shown that *P. cocos* repaired the intestinal barrier through reducing the abundance of pathogenic bacteria such as *Proteobacteria*, *Cyanobacteria*, *Helicobacteraceae*, and *Ruminococcaceae*, whereas increasing the number of beneficial bacteria such as *Bifidobacteriaceae* (Zou et al., 2021).

#### 5.3.2 Total flavonoids from Citrus aurantium L.

The records of TCM have shown that *C. aurantium* could effectively treat gastrointestinal disorders including diarrhea, bloating and abdominal pain (Kurita et al., 2000). Modern studies have shown that the total flavonoids of *C. aurantium* (CAF) had the effect of antioxidant, anticancer, anti-inflammatory (Maksoud et al., 2021). Liu et al. have found that CAF could significantly regulate the abundance and diversity of the gut microbiota of 5-FU-induced diarrhea in mice. At the phylum level, it increased the abundance of *Firmicutes*, decreased the abundance of *Bacteroidetes* and *Proteobacteria*, and improved the *Firmicutes*/*Bacteroidetes* (F/B) ratio. And it also reduce the number of pathogenic bacteria such as *Bacteroides*, and increased the abundance of beneficial bacteria *Lactobacillus*, therefore restoring 5-FU-induced intestinal dysbiosis, which improved intestinal mucosal damage and reduced the degree of diarrhea in mice (Danning et al., 2021).

#### 5.3.3 Dehydrodiisoeugenol

Dehydrodiisoeugenol (Figure 4G), a lignan isolated from *Myristica fragrans* Houtt., possesses multiple biological activities, including antitumor, antioxidant, anti-inflammatory, and hepatoprotective effects (Li and Yang, 2008). Research by Gao et al. demonstrated that dehydrodiisoeugenol (25 and 75 mg/kg) ameliorates CPT-11-induced intestinal mucositis by mitigating weight loss, colon shortening, epithelial barrier dysfunction, and the loss of goblet cells and intestinal stem cells. Furthermore, dehydrodiisoeugenol exhibited synergistic antitumor effects with CPT-11. The anti-CID effect of dehydrodiisoeugenol is dependent on the gut microbiota. Analysis of the gut microbiota in clinical patients and CID mice revealed a significant correlation between CPT-11-induced chemotoxicity and bacteria expressing β-glucuronidase, particularly *Enterococcus faecalis*. Dehydrodiisoeugenol treatment suppressed the CPT-11-induced increase in *E. faecalis*, consequently leading to reduced levels of β-glucuronidase and the toxic metabolite SN-38. The inhibitory effect of dehydrodiisoeugenol on CPT-11-induced intestinal toxicity was further validated using intestinal organoids. These results unveil a microbiota-driven, epithelial reconstruction-mediated mechanism of action for dehydrodiisoeugenol against CID (Gao et al., 2024).

#### 5.3.4 Dihydrotanshinone

Dihydrotanshinone (Figure 4H) is a main active metabolite of *Salvia miltiorrhiza* Bge., which has the effects of antibacterial, antioxidant, anti-inflammatory, and anticancer (Wang X. et al., 2020). The effect of dihydrotanshinone on gut microbiota composition was explored in the mice model of intestinal mucositis with the induction of 5-FU or CPT-11 by Wang et al. The results revealed that dihydrotanshinone could effectively improve the disorder of fecal microbiota structure, increased the abundance of the beneficial bacterium *Akkermansia*. And it also enriched bacterial species including *Ruminococcaceae*, *Lachnospiraceae*, which negatively correlated with the production of inflammatory factors in intestine, indicating that dihydrotanshinone alleviated CID probably through regulating the dysbiosis of gut microbiota (Wang L. et al., 2020).

#### 5.3.5 Berberine

The isoquinoline alkaloid, berberine (Figure 4I), abundant in Huanglian, has been used to treat diarrhea in clinical practice. Pharmacological studies have shown that it has the effects of antibacterial, anti-inflammatory, and anticancer (Zou et al., 2017). It was reported that berberine significantly regulated the structure and abundance of the gut microbiota in rats with CID induced by 5-FU. Meanwhile, it increased the abundance of Firmicutes and decreased the abundance of Proteobacteria. And it also enriched the number of beneficial bacteria, such as *Lactobacillus*, *Clostridium*, and *Ruminococcus*, and decreased the abundance of the pathogenic bacteria, including *Escherichia* and *Shigella*. These results indicated that berberine alleviated the diarrhea might through regulating the homeostasis of gut microbiota (Chen et al., 2020).

## 6 Discussion

CID is a prevalent and debilitating adverse effect experienced by cancer patients undergoing chemotherapy, significantly impacting treatment efficacy and patient quality of life. Despite the presence of pharmacological options like loperamide and octreotide, there remains a pressing need for more effective and safer interventions due to the limitations and adverse effects associated with these conventional treatments (Andreyev et al., 2014). Recent studies have highlighted the potential of TCM as a complementary approach to alleviate CID (Wang et al., 2019; Jiang et al., 2021). In this context, we comprehensively summarized the efficacy of TCM in managing CID and the underlying mechanisms, including suppression of enterocyte oxidative stress, inhibition of intestinal inflammatory responses, reduction of epithelial cell apoptosis, and modulation of gut microbiota dysbiosis (Table 1).

**TABLE 1:** The effects and mechanism of TCM on chemotherapy-induced diarrhea.

Name	Composition/Source	Experimental model	Dosage	Oxidative stress response	Inflammatory response	Cell proliferation and apoptosis	Gut microbiota structure	References
GGQLD	*P*. *lobata*, *S*. *baicalensis*, *C*. *chinensis*, and *G*. *uralensis*	Kunming mice/CPT-11	3.7, 1.85 g/kg	↑Keap1, ↑Nrf2, ↑HO-1, ↑GSH-PX	↓TNF-*α*, ↓IL-1*β*, ↓MPO	—	—	Wu et al. (2021)
HQD	*S*. *baicalensis*, *P*. *lactiflora*, *G*. *uralensis*, and *Z*. *jujuba*	BALB/c mice/5-FU	2.275, 4.550, 9.100 g/kg	↑Nrf2, ↑HO-1, ↑NQO1, ↑GSH-PX, ↑CAT, ↑SOD, ↓MDA	↓NF-*κ*B p65, ↓TNF-*α*, ↓COX-2	—	—	Li et al. (2022)
AEE	*A*. *sinensis*	Kunming mice/5-FU	200, 400, 800 mg/kg	↑SOD, ↓MDA	↓TNF-*α*, ↓COX-2	↑PCNA	—	Zheng et al. (2019)
BJO	*B*. *javanica*	Kunming mice/5-FU	0.125, 0.25, 0.50 g/kg	↑Nrf2, ↑HO-1, ↑SOD, ↓MDA	↓IL-1*β*, ↑IL-4, ↓TNF-*α* ↓IL-6, ↓iNOS, ↓COX-2, ↓CXCL1/2, ↓NLRP3	↑PCNA, ↓Bax, ↓Caspase-3, ↑Bcl-2	—	Zheng et al. (2023)
WMP	*P*. *mume*, *A*. *heterotropoides*, *Z*. *officinaie*, *C*. *chinensis*, *A*. *sinensis*, *A*. *carmichaelii*, *Z*. *bungeanum*, *C*. *cassia*, *P*. *ginseng*, *and P*. *chinense*	Balb/c mice/5-FU	11,325, 22,650 mg/kg	—	↓TLR4, ↓MyD88, ↓NF-*κ*B, ↓TNF-*α*, ↓IL-6, ↓IL-1*β*, ↓MPO	—	Regulate microbial composition and diversity, ↑*Firmicutes* ↓*Bacteroidetes*, ↓*Bacteroidia*, ↓*Clostridia*, ↑*Lactobacillaceae*, ↑*Bacteroidaceae*, ↓*Staphylococcales*, ↓*Muribaculaceae*, ↑SCAFs, ↑acetate, ↑propionate, ↑butyrate	Lu et al. (2022)
BPHWD	*A*. *membranaceus*, *A*. *macrocephala*, *C*. *aurantium*, *P*. *ternata*, MMF, *H*. *vulgare*, and *P*. *cocos*	Sprague Dawley rats/5-FU	10.5 mg/kg	—	↓TLR4, ↓NF-*κ*B, ↓TNF-*α*, ↓IL-1*β*, ↓IL-6	—	—	Sun et al. (2019)
SBWSGCR	*B*. *chinense*, *P*. *frutescens*, *C*. *aurantium*, *P*. *heterophylla*, *A*. *macrocephala*, *P*. *cocos*, *M*. *officinalis*, *E*. *brevicornu*, *C*. *orchioides*, *R*. *laevigata*, and *A*. *lappa*	Tumor-bearing BALB/c mice/CPT-11	100, 200 mg/kg	—	↓TLR4, ↓NF-*κ*B, ↓IL-1*β*, ↓IL-18, ↓TNF-*α*	—	—	Zhang et al. (2019)
RABD	*P*. *ginseng*, *A*. *macrocephala*, *P*. *cocos*, *G*. *uralensis*, *C*. *reticulata*, *P*. *ternata*, *A*. *lappa*, *and A*. *villosum*	BALB/c mice/5-FU	0.3, 1, 3 g/kg	—	↓NF-*κ*B p65, ↓IL-6, ↓IL-1*β*, ↓TNF-*α*, ↓COX-2	—	—	Liu et al. (2021a)
*M*. *officinalis*	*M*. *officinalis*	Kunming mice/CPT-11	400, 800 g/kg	↑SOD, ↓MDA	↓TLR4, ↓NF-*κ*B, ↓IL-1*β* ↓IL-6, ↓TNF-*α*, ↓COX-2, ↓iNOS	—	—	Xia (2019)
Isoliquiritigenin	*G*. *uralensis*	BLAB/c mice/5-FU	117, 234 μmol/kg	—	↓NF-*κ*B p65, ↓PTGS2, ↓NOS2, ↓TNF-*α*	—	—	Liao et al. (2022)
Puerarin	*P*. *lobata*	C57BL/6 mice/5-FU	50, 75, 100 g/kg	↑GSH, ↓MDA, ↑SOD	↓JAK2, ↓STAT3, ↓SOCS3, ↓COX-2, ↓iNOS, ↓IL-1*β*, ↓IL-6, ↓TNF-*α*	↓Bax, ↑Bcl-2	—	Wang et al. (2021)
Cinobufotalin	*B*. *gargarizans*	BLAB/c mice/FOLFOX (5-FU, oxaliplatin and calcium folinate)	260, 520 mg/kg	—	↓NLRP3, ↓NF-*κ*B, ↓IL-6, ↓IL-1*β*, ↓TNF-*α*	—	—	Li et al. (2022)
HLJDD	*C*. *chinensis*, *P*. *chinense*, *G*. *jasminoides*, and *S*. *baicalensis*	Mice/5-FU/CPT-11	50, 100,200 mg/kg	—	—	↑Wnt, ↓TUNEL, ↓Caspases-3, ↑Ki67, ↑CD44, ↑lgr5, ↑ascl2, ↑oflm4, ↑Wnt3, ↑Axin2, ↑Fzd5 ↑Pygo2	—	Chan et al. (2020)
QJFZG	*S*. *diffusum*, *H*. *vulgare*, *A*. *memeranaceus*, and *S*. *barbata*	CT-26 tumor-bearing xenograft mice/5-FU	2 g/kg	—	↓TNF-*α*, ↓IL-1*β*, ↓IL-6, ↑IL-10	↓TUNEL, ↑Bcl-2 ↓Bax, ↑PCNA, ↑Cyclin D1, ↑CKD4, ↓p21	—	Zhang et al. (2019)
RBG	MMF, *S*. *cuneata*, and *H*. *vulgare*	BLAB/c mice/5-FU	150 mg/mL	—	—	↑AKT, ↓TUNEL, ↓Bax, ↑Bcl-2, ↑PCNA, ↑CKD4, ↑c-myc	—	Li (2020)
PCCL	*C*. *chinensis*	C57BL/6 mice/5-FU	20 mg/kg	—	↓TNF-*α*, ↓IL-6, ↓IL-1*β*	↓TUNEL, ↓Caspases-3, ↓Bax, ↑Bcl-2	—	Ji et al. (2022)
Andrographolide	*A*. *paniculata*	BALB/c mice/5-FU	25, 50, 100 mg/kg	—	↓TNF-*α*, ↓IL-1*β*, ↓IL-6	↓p38 MAPK, ↓TUNEL, ↑PCNA, ↓Bax, ↑Bcl-2, ↓Caspases-8, ↓Caspases-3	—	Xiang et al. (2020)
BXXXD	*P*. *ternate*, *Z*. *officinale*, *C*. *chinensis*, *S*. *baicalensis*, *P*. *ginseng*, *Z*. *jujuba*, and *G*. *uralensis*	Sprague Dawley mice/5-FU	2.5 g/kg	↓MDA, ↑SOD	—	—	Regulate the structure and abundance of intestinal flora, ↑*Firmicutes*, ↑*Patescibacteria*, ↓*Bacteroidetes*	He et al. (2023)
SJXXD	*Z*. *officinale*, *C*. *chinensis*, *S*. *baicalensis*, *P*. *ternata*, *G*. *uralensis*, *Z*. *jujuba*, and *C*. *pilosula*	Sprague Dawley mice/CPT-11	2 g/mL	—	↓TLR4, ↓MyD88, ↓JNK, ↓NF-*κ*B, ↓IL-1*β*, ↓IL-6, ↓TNF-*α*, ↑IL-10, ↑TGF-*β*1	—	Regulate the microbiota structure and abundance ↑*Bacteroidota*, ↑*Muribaculaceae*, ↑*Bacteroides*, ↑*Akkermansia*, ↑SCFAs	Gao (2022)
*P. cocos*	*P*. *cocos*	C57BL/6 mice/Cisplatin	2.0 g/kg, 7.6 mg/kg	—	↓IL-2, ↓IL-6, ↓TNF-*α*	—	Restore the microbiota community, ↓*Proteobacteria*, ↓*Cyanobacteria*, ↓*Helicobacteraceae*, ↓*Ruminococcaceae*, ↑*Bifidobacteriaceae*	Zou et al. (2021)
CAF	*C*. *aurantium*	BALB/c mice/5-FU	50, 100, 200 mg/kg	↑SOD ↑GSH-PX ↓MDA	↓TNF-*α*, ↓IL-1*β*, ↓IL-6	—	↑diversity and abundance of intestinal flora, ↑*Firmicutes*, ↓*Bacteroidetes*, ↓*Proteobacteria*, ↑F/B, ↓*Bacteroides*, ↑*Lactobacillus*	Liu et al. (2021b)
Dihydrotanshinone	*S*. *miltiorrhiza*	C57BL/6 mice/5-FU/CPT-11	10 mg/kg	—	↓IL-6, ↓TNF-*α*	—	Restore disordered fecal microflora, ↑*Akkermansia*, ↑*Ruminococcaceae*, ↑*Lachnospiraceae*	Wang et al. (2020a)
Berberine	*C*. *chinensis*	Sprague Dawley rats/5-FU	100 mg/kg	—	↓IL-1*β*, ↓IL-6, ↓TNF-*α*	—	Regulate diversity and community composition of gut microbiota in feces, ↑*Firmicutes*, ↓*Proteobacteria*, ↑*Lactobacillus*, ↑*Clostridium*, ↑*Ruminococcus*, ↓*Escherichia*/*Shigella*	Chen et al. (2020)

A hallmark of TCM in treating CID lies in its multidimensional regulatory capacity, which transcends the single-target approach of conventional antidiarrheal agents (e.g., loperamide). Unlike monotherapies that merely suppress symptoms, TCM leverage holistic theory to synchronously target interconnected pathological axes, thereby presenting a more comprehensive and effective therapeutic paradigm (Wang X. et al., 2024).​ In terms of the concurrent modulation of multiple pathways, WMP serves as a typical example. It can simultaneously exert multiple therapeutic effects. On one hand, it suppresses inflammation by inhibiting the TLR4/MyD88/NF-κB signaling pathway, resulting in decreased levels of proinflammatory cytokines such as TNF-α and IL-6 (Lu et al., 2022). On the other hand, it restores the balance of the gut microbiota, specifically increasing the ratio of *Firmicutes*/*Bacteroidetes* and the production of SCFAs (Lu et al., 2024). Additionally, it enhances the intestinal barrier function by upregulating the expressions of tight junction proteins like ZO-1 (Lu et al., 2022). Similarly, HQD also exhibits concurrent regulatory effects. It inhibits ferroptosis through the p53/GPX4 pathway, therefore repaired the intestinal barrier function (Wang et al., 2025). It also ameliorates gut microbiota dysbiosis and suppresses the activity of bacterial β-glucuronidase, thereby inhibiting the generation of toxic SN-38 and ultimately alleviating CID (Teng et al., 2024). Another example is BJO, which integrates Nrf2-driven antioxidation with cGAS-STING-mediated immunomodulation, concurrently alleviating mucosal injury and diarrhea (Zheng et al., 2023; Lai et al., 2025). Such holistic regulation and multi-targeted actions enable TCM to address the complex pathological processes of CID in a coordinated manner, leading to better clinical outcomes compared to traditional single-target therapies. The cases of WMP, HQD, and BJO illustrate how TCM formulations can simultaneously target inflammation, gut microbiota, and barrier function in experimental models of CID. This coordinated, multi-targeted action suggests better clinical outcome compared to traditional single-target therapies.

Critically, the four primary mechanisms of TCM in treating CID, including antioxidation, anti-inflammation, barrier maintenance, and microbiota regulation, engage in bidirectional crosstalk, forming a coordinated regulatory network. Oxidative stress further augments the aggressiveness of inflammation. Study has shown that chemotherapy-induced ROS activate inflammasome, which in turn triggers the release of proinflammatory cytokines, ultimately disrupting the tight junctions of the intestinal epithelium (Jang et al., 2025). Concurrently, microbiota dysbiosis amplifies inflammation and intestinal barrier damage. Studies have shown that a reduction in SCFAs impairs synthesis of mucin and antimicrobial peptides, exposing the epithelium to pathogens, which then increase the proportion of pro-inflammatory immune cells and activate the TLR4/NF-κB pathway, and finally propagate inflammation (Liu P. et al., 2021). Conversely, barrier repair can mitigate microbial toxicity. Studies have shown that the upregulation of ZO-1 and Claudin-1 induced by TCM prevents bacterial translocation, thereby reducing microbiota mediated drug reactivation (Dahlgren and Lennernas, 2023). This intricate cross-talk ensures that the regulatory effects of TCM are not isolated but mutually reinforcing, enhancing the overall therapeutic efficacy.

In recent years, TCM has gained increasing attention as an adjuvant therapy for alleviating CID, owing to its observed clinical benefits and multi-target therapeutic characteristics. The literature summarized in this review provides valuable insights into the mechanisms of TCM in CID. However, several limitations must be acknowledged to objectively evaluate its therapeutic potential. Through our systematic review of the literature, we observed that while TCM demonstrates encouraging clinical outcomes for CID, the evidence is often constrained by methodological limitations. Common issues include inadequate randomization, insufficient blinding procedures, and small sample sizes across many of the studied clinical trials. Moreover, in the context of cancer treatment—where polypharmacy is common—the potential risk of botanical drug-drug interactions also raises concerns regarding safety and efficacy. These challenges are compounded by significant variability in TCM formulations, extraction methods, and dosages across studies, which complicates the reproducibility and standardization of treatments. Meanwhile, regulatory challenges and a lack of universally accepted quality control standards hinder the global acceptance and integration of TCM into conventional oncology care. Future efforts should focus on conducting well-controlled clinical trials, standardizing TCM formulations, and systematically evaluating safety profiles to facilitate its evidence-based application in CID management. In summary, while TCM holds significant promise as a multi-target therapy for CID, its successful translation into clinical practice will depend on rigorous scientific validation, international regulatory cooperation, and standardized practice.

## Glossary

ROS: Reactive oxygen species
RNS: Reactive nitrogen species
GSH-PX: Glutathione peroxidase
CAT: Catalase from *micrococcus* lysodeiktic
SOD: Superoxide dismutase
Keap1: Kelch like ECH associated protein 1
Nrf2: Nuclear factor erythroid 2 related factor 2
NQO1: NADPH quinone dehydrogenase 1
HO-1: Heme oxygenase 1
MDA: Malondialdehyde
TNF-α: Tumor necrosis factor-α
IL-6: Interleukin-6
IL-1β: Interleukin-1β
COX-2: Cyclooxygenase-2
TLRs: Toll-like receptors
NF-*κ*B: Nuclear factor-*κ*B
IL-10: Interleukin-10
TLR4: Toll-like receptor 4
MyD88: Myeloid differentiation factor 88
ZO-1: Zonula occluden 1
NLRP: NOD-like receptor thermal protein domain associated protein
IL-18: Interleukin-18
iNOS: Inducible nitric oxide synthase
PTGS2: Prostaglandin-endoperoxide synthase 2
NOS2: Nitric oxide synthase 2
JAK2: Janus kinase 2
SOCS3: Suppressor of cytokine signaling 3
DAO: Diamine oxidase
ET: Endotoxin
D-LA: D-lactic acid
PKB (AKT): Protein kinase B
MAPK: Mitogen-activated protein kinase
Caspase: Cysteinyl aspartate specific proteinase
Bcl-2: B-cell lymphoma-2
Bax: Bcl-2 associated X
Lgr5: Leucine repeat G protein-coupled receptor 5
PCNA: Proliferating cell nuclear antigen
CDK4: Cyclin-dependent kinase 4
c-Myc: Cellular-myelocytomatosis viral oncogene
SCFAs: Short chain fatty acid
F/B: *Firmicutes*/*Bacteroidetes*
STAT3: Signal transducer and activator of transcription 3
